# Daily lives of university students in the health area during the beginning of the Covid-19 pandemic in Brazil

**DOI:** 10.17533/udea.iee.v39n3e07

**Published:** 2021-11-08

**Authors:** Luciana Regina Ferreira da Mata, Juliana Dias Reis Pessalacia, Tatiane Prette Kuznier, Priscila Kelly da Silva Neto, Caroline de Castro Moura, Fernando Ribeiro dos Santos

**Affiliations:** 1 Nurse, Ph.D. Associate Teacher. Federal University of Minas Gerais, Brazil. Email: lucianamata@ufmg.br (Corresponding author). Universidade Federal de Minas Gerais Federal University of Minas Gerais Brazil lucianamata@ufmg.br; 2 Nurse, Ph.D. Associate Teacher. Federal University of Mato Grosso do Sul, Brazil. Email: juliana.pessalacia@ufms.br Universidade Federal de Mato Grosso do Sul Federal University of Mato Grosso do Sul Brazil juliana.pessalacia@ufms.br; 3 Nurse, Ph.D. Adjunct Teacher. Federal University of Paraná, Brazil. Email: tatianeprette@gmail.com Universidade Federal do Paraná Federal University of Paraná Brazil tatianeprette@gmail.com; 4 Nurse, Master’s Student. State Department of Health of Mato Grosso do Sul, Brazil. Email: priscila.baldonado@gmail.com State Department of Health of Mato Grosso do Sul Brazil priscila.baldonado@gmail.com; 5 Nurse, Ph.D. Adjunct Teacher. Federal University of Viçosa, Brazil. Email: caroline.d.moura@ufv.br Universidade Federal de Viçosa Federal University of Viçosa Brazil caroline.d.moura@ufv.br; 6 Undergraduate Medical Student. Federal University of Mato Grosso do Sul, Brazil. Email: fernanndoribeiro@hotmail.com Universidade Federal de Mato Grosso do Sul Federal University of Mato Grosso do Sul Brazil fernanndoribeiro@hotmail.com

**Keywords:** COVID-19, students, health occupations, higher education institutions**.**, COVID-19, estudiantes del área de la salud, instituciones de enseñanza superior**.**, COVID-19, pandemias, estudantes de ciências da saúde, instituições de ensino superior

## Abstract

**Objective:**

To determine the main changes that took place in the daily lives of students in the health area during the beginning of the Covid-19 pandemic in Brazil.

**Method:**

This is a cross-sectional study, carried out from May to June 2020, with 1786 students over 18 years old, regularly enrolled in health courses at higher education institutions in five regions of Brazil. Sampling was by convenience, typified as snowball. In order to collect data, an instrument to describe the sociodemographic profile and the daily lives of students during the pandemic period was used, which was applied via a digital platform on the web.

**Results:**

The main changes that took place in the daily lives of academic students in the health area in the face of the Covid-19 pandemic in Brazil are related to lower productivity; difficulty concentrating; increased hours of sleep, use of electro-electronic equipment and weight; poorer quality of food; higher consumption of food, legal and illegal substances and medications (mainly analgesics, anxiolytics and antidepressants); less interest in personal appearance; and greater contact with relatives. Regarding emotional changes, it should be underlined the complaints of anxiety, stress, anguish, confusion, helplessness and depression.

**Conclusion:**

During the beginning of the Covid-19 pandemic in Brazil, the students in the health area experienced several changes in their daily lives, which deserve special attention from higher education institutions and health systems, envisioning interventions to minimize health risks to this population.

## Resumen

**Objetivo:**

Determinar los principales cambios ocurridos en la vida cotidiana de los estudiantes del área de la salud durante el inicio de la pandemia de COVID-19 en Brasil**.**

**Métodos:**

Estudio transversal realizado a 1786 estudiantes mayores de 18 años, regularmente matriculados en cursos del área de la salud en instituciones de enseñanza superior en cinco regiones de Brasil. El muestreo fue por conveniencia del tipo bola de nieve. Para la recolección de datos se utilizó un instrumento para describir el perfil sociodemográfico y la rutina de los estudiantes al inicio del periodo de la pandemia, el cual se aplicó a partir de una plataforma digital en la web.

**Resultados:**

Los principales cambios ocurridos diariamente están relacionados con la menor productividad; la dificultad de concentración, el aumento en las horas de sueño, del peso y el uso de dispositivos electrónicos, la inadecuada alimentación, el mayor consumo de alimentos, el uso sustancias lícitas e ilícitas y medicamentos (principalmente analgésicos, ansiolíticos y antidepresivos), el menor interés por la apariencia personal y el menor contacto con los familiares. En cuanto a los cambios emocionales, se presentó en mayor medida ansiedad, estrés, angustia, confusión, impotencia y depresión**.**

**Conclusión:**

Los estudiantes de salud durante el inicio de la pandemia de COVID-19 en Brasil experimentaron varios cambios en su vida cotidiana, que merecen una atención especial por parte de las instituciones de educación superior y los sistemas de salud, con el objetivo de realizar intervenciones para minimizar los riesgos para la salud de esta población**.**

## Resumo

**Objetivo:**

Determinar as principais mudanças ocorridas no quotidiano de estudantes da área de saúde durante o início da pandemia da Covid-19 no Brasil.

**Métodos:**

Estudo transversal, realizado no período de maio a junho de 2020 com 1786 estudantes com mais de 18 anos, regularmente matriculados em cursos da área da saúde em instituições de nível superior de cinco regiões do Brasil. A amostragem foi por conveniência, do tipo bola de neve. Para coleta de dados, utilizou-se um instrumento para descrever o perfil sociodemográfico e a rotina dos estudantes durante o período de pandemia, que foi aplicado via plataforma digital na web.

**Resultados:**

As principais mudanças ocorridas no quotidiano dos acadêmicos da área da saúde frente à pandemia da Covid-19 no Brasil estão relacionadas à menor produtividade; dificuldade de concentração; aumento de horas de sono, do peso, de uso de equipamentos eletroeletrônicos; pior qualidade da alimentação; maior consumo de alimentos, substâncias lícitas e ilícitas e medicamentos (principalmente analgésicos, ansiolíticos e antidepressivos); menor interesse pela aparência pessoal; e maior contato com familiares. Em relação às alterações emocionais, destacam-se maiores queixas de ansiedade, estresse, angústia, confusão, impotência e depressão.

**Conclusão:**

Os estudantes da área de saúde durante o início da pandemia da Covid-19 no Brasil vivenciaram diversas mudanças no quotidiano, as quais merecem atenção especial por parte das instituições de ensino superior e dos sistemas de saúde, vislumbrando-se intervenções para minimizar os riscos à saúde desta população

## Introduction

Everyday life refers to the way of life of people and the community, being represented by interactions, values, beliefs, symbols and images that outline the way of living healthy or acquiring diseases.(1) In the context of the Coronavirus Disease 2019 (COVID-19) pandemic, the daily lives of university students changed after the interruption of face-to-face activities by 91% of them around the world.(2) Considering non-pandemic periods, entering university already brings psychological implications for students. It is a period of formation of new relationship cycles, with people from different contexts and social experiences. The academic trajectory is permeated with different challenges related to the involvement with a new learning universe, which requires skills and competencies not yet experienced, which may trigger anxiety and insecurity.(3)

During the COVID-19 pandemic, the daily lives and usual interactions of students were impacted by social distancing measures as a strategy to control the spread of the Coronavirus.(4) Nevertheless, such changes have generated emotional impacts across the entire population.(5) In the academic context, face-to-face classes have been replaced by classes in digital media, based on Information and Communication Technologies (ICTs).(2) In this sense, the integrity of the curricular program has become vulnerable to the consequences imposed by COVID-19, since, in health courses, clinical practice is a central element in training. In addition, the lack of psychological support, the work overload of teachers and students, the lack of quality in teaching resulting from the absence of planning of activities by ICTs and access to these technologies have impacted daily life and genetared dissatisfaction in students during the pandemic period.(2)

In this context, changes in the daily lives of university students can generate biopsychosocial alterations that affect their health. Therefore, studies that seek to investigate new routines and modes of interaction of students in the health area become relevant, in a scenario unknown to the population.

Accordingly, this study has the objective of knowing and analyzing what were the main changes that took place in the daily lives of academic students in the health area during the beginning of the period of the COVID-19 pandemic in Brazil, in order to understand the impacts of the pandemic on the biopsychosocial aspects of these individuals.

## Methods

Quantitative, cross-sectional and descriptive study. Data collection was conducted from May 19, 2020 to June 3, 2020, which characterizes the early period of the pandemic in Brazil. This study was attended by 1786 students enrolled in the health area courses in higher education institutions in the five regions of Brazil. Inclusion criteria were: being over 18 years old and regularly enrolled in a health course. Participants who did not live in the country or who mentioned they had dropped out before the pandemic began were excluded. Sampling was by convenience, typified as snowball, since the researchers asked the participants to forward the link or the reference of potential participants who met the inclusion criteria.

The data collection instrument was designed by the researchers based on two literatures that supported the definition of variables related to the investigation of the sociodemographic profile and the routine of students during the pandemic period.(6,7) Therefore, the following variables were included: gender; age range; region of residence in the country; nature of the educational institution (if public or private); course; period; shift; practice of social distancing; sleep patterns, productivity, mood and diet during the pandemic period; changes in weight and interest in personal appearance; family and social interaction; changes in the pattern of use of licit and/or illicit substances; and emotional changes resulting from the pandemic period of COVID-19.

The instrument was evaluated regarding the content and organization of the items that composed each question by five nurse teachers, PhDs in nursing, two specialists in the area of mental health and three in the area of adult health. After the suggested adjustments, the instrument with 15 questions was inserted into *Google Forms*®, a digital platform available for free on the web. In order to collect data, the link to access the instrument along with the free and informed consent form was sent to the participants via email and/or *Whatsapp*®. The average time of completion was seven minutes. The data were exported to *Microsoft Excel*®, version 2013, for organization and coding of variables. In order to perform the descriptive analysis, the *Statistical Package for the Social Sciences* (SPSS) statistical software, version 20, was used. Each item of the instrument was processed as a categorical variable and the results were presented as absolute and relative frequencies.

The study was approved by an ethics committee for research with human beings (CAAE nº 31550820.0.0000.5620/Opinion Number: 4.021.458), which followed the recommendations of Resolution 466/2012 of the National Health Council.(8)

## Results

Of the 1786 students who took part in this study, 83.6% were female. Most were between 18 and 22 years old and lived in the Southeast region of the country (Table 1).

Regarding the institution, 63.7% of the students attended a public university; and 54.2% studied nursing. In addition, 69% were in the early cycle of the course (from the first to the third year). Concerning the shift, 65.4% studied full-time.

**Table 1:** General characteristics of the study sample (*n*=1786)

Variables	*n*	%
Female		
Female	1493	83.6
Male	293	16.4
Age range in years		
18-22	1128	63.2
23-27	439	24.5
28-32	107	6.0
>33	112	6.3
Region of residence		
Southeast	804	45.0
South	490	27.4
Midwest	280	15.7
Northeast	150	8.4
North	62	3.5
Type of university		
Public	1137	63.7
Private	649	36.3
Courses		
Nursing	968	54.5
Medicine	368	20.7
Pharmacy	124	7.0
Nutrition	86	4.8
Dentistry	51	2.9
Physiotherapy	42	2.4
Biomedicine	38	2.1
Biochemistry	32	1.8
Occupational Therapy	20	1.1
Others	48	2,7
Shift		
Full-time	1168	65.4
Evening	345	19.3
Morning	258	14.4
Afternoon	15	0.8

It was also found that 93.6% of the students reported that they were practicing social distancing. However, 76.7% had difficulty in complying with it. As for sleep patterns, 90.8% of the students reported that they had changed, with 79.5% sleeping more. A change in activity patterns was also observed in 94.4% of the students, with 73.3% reporting lower productivity (Table 2).

Regarding the practice of leisure activities, only 1.8% of the participants stated that there was no change, and most of them started to use the internet more and watch more television/movies. The dietary patterns and weight of these students also changed in this early period of the COVID-19 pandemic, as 87.0% reported that the quality of diet worsened and 46.5% reported increased body weight. Students also perceived changes in interest in personal appearance, so that 70.8% reported taking less care of their appearance (Table 2).

**Table 2:** Routine of social distancing, changes in sleep patterns, activity, leisure, diet, body weight and interest in personal appearance of the 1786 students during the early period of the Coronavirus pandemic

Variables	*n*	%
Routine of social distancing*		
Change in routine	1740	97.4
Leaves home to go shopping	1340	75.0
Isolated without leaving home	524	29.3
Leaves home to work	323	18.1
Leaves home to practice physical and/or leisure activities	138	7.7
Leaves home to practice voluntary actions	79	4.4
Changes in sleep patterns		
Sleep more	1420	79.5
Sleep less	202	11.3
Without changes	164	9.2
Changes in activity patterns*		
Lower productivity	1310	73.3
Less mood	899	53.3
Difficulty concentrating	787	46.7
Without changes	100	5.6
Leisure activities*		
Internet/Social networks	1477	82.7
Television/Movies	1249	69.9
Music	806	45.1
Cooking	691	38.7
Reading	570	31.9
Electronic/online games	384	21.5
Photo production/editing	253	14.2
Contact with friends/relatives	160	9.0
Video production	122	6.8
Without changes	33	1.8
Changes in dietary patterns*		
Quality of food has worsened	1553	87.0
Need to eat more often	1048	58.7
Without changes	307	17.2
Changes in body weight		
Weight gain	831	46.5
Weight reduction	228	12.8
Kept the usual weight	484	27.1
Did not know how to inform	243	13.6
Interest in personal appearance		
Take less care of my appearance in general	1265	70.8
Keep the same routine	521	29.2

* The participant may have marked more than one answer option.

Table 3 presents information about family and social interaction during the pandemic period. It can be seen that most students stayed close to their relatives.

**Table 3:** Family and social interaction of the 1786 students during the early stage of the Coronavirus pandemic

Variables	*n*	%
Family interaction		
Without changes	333	18.6
Closer to family, and the relationship is the same	652	36.5
Closer to family, and the relationship has improved	334	18.7
Closer to family, and the relationship has worsened	231	12.9
Physically distant from family, and the relationship is the same	181	10.1
Physically distant from family, and the relationship has improved	32	1.8
Physically distant from family, and the relationship has worsened	23	1.3
Social interaction*		
Increased contact with relatives	1320	73.9
Increased contact with friends	1153	64.6
Religious/spiritual practices	318	17.8
Volunteer actions	101	5.7

* The participant may have marked more than one answer option.

Changes in the pattern of consumption of licit and/or illicit substances were also observed. A percentage of 65.5% of the students did not use these substances; however, an increase of 19% was observed in the consumption of alcohol, 5.1% in the consumption of cigarettes, and 2.3% in the consumption of illegal drugs. Moreover, an increase was observed in the consumption of drugs according to medical prescription (7.8%) and on their own (7.2%), especially anxiolytics (11.7%), analgesics (11.5%) and antidepressants (11.2%).

Finally, Table 3 presents the emotional changes that took place in students during the pandemic period. 

**Table 4:** Emotional changes arising from the Coronavirus pandemic period (n=1786)

Variables*	*n*	%
Anxiety	1241	69.5
Stress	1139	63.8
Anguish	1079	60.4
Confusion	945	52.9
Helplessness	879	49.2
Depression	700	39.2
Without perspective on the future	396	22.2
Desire to take one’s own life	170	9.5

* The participant may have marked more than one answer option.

## Discussion

From the analysis of the daily life of health academic students during the early period of the COVID-19 pandemic in Brazil, the main changes/effects observed in the routine of the surveyed students were: social distancing and the difficulty in complying with it; increased sleep time; lower productivity, less mood and difficulty concentrating; increased use of the internet and television/movies; increased consumption of low-quality food; less care with appearance; increased contact and interaction with relatives; increased consumption of alcohol and medications, mainly, anxiolytics, analgesics and antidepressants; and increased sensations of anxiety, stress, anguish, confusion, helplessness and depression.

The analysis of the changes in the daily lives of students indicates that the social distancing measures and the remote teaching implemented in Brazilian Higher Education Institutions (HEIs) impacted on different biopsychosocial aspects of these students, which deserve more attention from managers in the formulation of policies and measures aimed to minimize the risks to the physical and emotional health of academic students. Most students reported adherence to the practice of social distancing; however, they pointed out difficulties in complying with it. A study(9) found that the difficulties in complying with social distancing are associated with the social position, the function and the work performed, which, in turn, define the living conditions of the population.(10) The quality of information, the credibility of government officials and the uncertainty about the virus are also factors that impact on adherence to social distancing.(10)

Most students reported a change in sleep patterns, with reports of increased hours of sleep. Research(11) conducted in Greece with university students in the midst of the COVID-19 pandemic found that the amount of sleep time increased, but that sleep quality worsened. Scholars point out that worsening sleep quality is due to subjective sleep quality, latency, duration, efficiency, sleep disturbances, sleep medication use and daytime dysfunction.(12) A survey(12) conducted with college students in China found that the severity of the COVID-19 outbreak can significantly increase people’s negative emotions and, consequently, decline in sleep quality. Also in China, among 939 individuals evaluated, sleep disturbances increased significantly in people aged 18 to 24 years, with 36.43% of participants reporting severely compromised sleep quality.(13)

In the present study, changes in the activity patterns were also highlighted, with reports of lower productivity, less mood and difficulty concentrating. Among Saudi Arabian medical students, difficulty concentrating and lower productivity during the quarantine period were also evidenced.(14) With regard to leisure habits, most students reported more time spent on the internet, social networks and television/movies. They also stated that they had increased the frequency of reading during the pandemic period. Research(15) conducted in Africa with 678 individuals aged 14 to 74 years evaluated the psychological impact of confinement linked to the COVID-19 epidemic and found that respondents made use of the Internet several hours a day, with greater interest in social media content such as *Facebook*, *Twitter* and *YouTube*. In fact, as a result of the COVID-19 pandemic, daily activities such as work and education started to be carried out online(5), which intensified contact by digital means compared to the pre-pandemic period.(16)

Another relevant finding was in relation to the quality of the diet of these students, which showed increased consumption of food products with poor nutritional quality and increased body weight. A survey(17) involving 1097 adults in Poland, conducted during the early period of the pandemic with the objective of evaluating the nutritional and consumption habits, indicated that most participants started to eat more, and these trends were more frequent in overweight and obese individuals, respectively. Increased Body Mass Index (BMI) was associated with less frequent consumption of vegetables, fruits and legumes and higher consumption of meat, dairy and fast food.(17) In Australia(18), the effects of social distancing measures on the dietary patterns of university students during the early phase of the COVID-19 pandemic were evaluated; and, among women, caloric intake was 20% higher. The frequency of snacks and energy density of these students increased during the pandemic when compared to the food consumption of the same population in the years 2018 and 2019. The longer time spent at home can encourage the consumption of high-calorie diets, with the increase in the number and meal portions.(18)

Students in the present study also reported changes in interest in personal appearance, so that most reported taking less care of their appearance. No studies with similar results were identified. Nevertheless, it is known that the increased use of videoconferencing technologies in the context of social distancing may indirectly contribute to increased concern with personal appearance. The current requirement for online self-image publications is shown to be detrimental to body image and mood in this population. The analysis of the effects of observing one’s own image during a conversation using digital technologies becomes an area of interest to be investigated.(19)

With regard to family and social interaction during the pandemic period, it is noted that most students became closer to their relatives. The physical closeness between people who live together may increase during the period of social distancing, specifically the time spent with children, partners, parents or siblings. This may have led to closer ties and the possibility of more quality time, which may strengthen some relationships, with increased complicity and emotional closeness. However, for others, there may be an emotional distancing, since this situation may exacerbate latent family conflicts, potentially aggravated by financial imbalances, unemployment or fear of losing a job.(16) In this sense, in Saudi Arabia, students admitted greater emotional detachment from family, partners and friends during the period of social distancing.(14)

In this study, it was observed changes in the pattern of consumption of legal and/or illegal substances, where, although the majority did not use these substances, among the participants who said they did, there was an increase in the consumption of alcohol, cigarettes, illegal drugs, self-medication, especially anxiolytics, analgesics and antidepressants. In a survey(20) that included 939 university students from Russia and Belarus, rates of substance use increased from the month before the pandemic, with increased use of tobacco, alcohol, marijuana, ritalin or similar substance, analgesics and sedatives. Russian and Belarusian students in quarantine had a significantly higher rate of alcohol use than those not restricted.(20) In Poland, researchers highlighted increased an alcohol consumption in adults and an increased frequency of smoking during the quarantine period.(17)

It was also noted that participants reported a greater propensity to negative emotional changes, such as anxiety, stress, depression and suicidal thoughts, which impacts their mental health. Studies conducted in Portugal,(9) Greece,(11) Saudi Arabia,(14) and China(21,22) showed an increase in depressive symptoms in university students during the pandemic period. There was an increase in symptoms related to anxiety (6,11,12,21,22) stress or post-traumatic stress disorder,(6,21), as well as an increase in suicidal thoughts.(11) Reception of negative information about the pandemic makes students more prone to anxiety symptoms and mild depression.(21) It is recommended greater involvement of relatives, friends and teachers to help them face negative emotions and difficulties with positive attitudes and increased social support.(21)

Lack of masks, alcohol and other products relevant to virus prevention and control have also been highlighted as factors related to increased concern on the part of students about the threat to life and health posed by COVID-19, which potentiates overall psychological distress and somatic symptoms.(22) In China, an investigation conducted with 805 undergraduate students identified that increased symptoms affecting the mental health of these students are associated with doubts about the potential negative impact that this period could have on academic progress.(4)

The limitation of this study is the selection of participants by the convenience sampling method, which generated a distribution of a larger number of participants from the Southeast region of Brazil, which does not guarantee the representativeness of the results for the entire Brazilian population. Another limitation was the non-use of a validated instrument for data collection, since there was no instrument in the literature that met the study objectives. Nevertheless, in order to minimize biases related to the reliability and validity of the instrument, it was submitted to the evaluation of five teachers with experience in the topic.

## Conclusion

The main changes that took place in the daily lives of health academic students facing the COVID-19 pandemic in Brazil are related to lower productivity; difficulty concentrating; increased hours of sleep, use of electro-electronic equipment and weight; poorer quality of food; higher consumption of food, legal and illegal substances, as well as medications (mainly analgesics, anxiolytics and antidepressants); less interest in personal appearance; and greater contact with relatives. Regarding emotional changes, it should be underlined the complaints of anxiety, stress, anguish, confusion, helplessness and depression. Such changes deserve special attention from HEIs and health systems, in order to direct interventions to minimize health risks to this population.

It is emphasized the importance of new studies that also aim to evaluate the daily lives of students at different times of the pandemic, in order to broaden the discussion about changes and maintenance of these behaviors among students. Such discussions will foster interventional studies with actions aimed to minimize the impacts of epidemic periods on the daily lives of students.
